# Integrated Single-Cell Profiling Reveals TL1A as a Biomarker and Driver of Type 2 Inflammation via Macrophage-Dependent Immunoregulation in Asthma

**DOI:** 10.34133/research.1190

**Published:** 2026-04-09

**Authors:** Jintao Zhang, Xiaofei Liu, Qian Qi, Yun Pan, Rong Zeng, Chenxiao Qiao, Changjuan Xu, Pengfei Wang, Shuochuan Shi, Ying Wang, Xuemin Liu, Liang Dong

**Affiliations:** ^1^ Department of Respiratory, The First Affiliated Hospital of Shandong First Medical University & Shandong Provincial Qianfoshan Hospital, Shandong Institute of Respiratory Diseases, Featured Laboratory of Respiratory Immunology and Regenerative Medicine in Universities of Shandong, Jinan Clinical Research Center for Respiratory Disease, Jinan, China.; ^2^ Department of Respiratory, Shandong Qianfoshan Hospital, Cheeloo College of Medicine, Shandong University, Jinan, China.

## Abstract

**Background:** Asthma remains a global health burden, with its heterogeneity necessitating precision biomarkers and targeted therapies. Tumor necrosis factor-like ligand 1A (TL1A), a novel alarmin in the airway, remains poorly characterized in asthma pathogenesis. **Methods:** TL1A levels were measured in the sputum and serum samples of patients with various asthma phenotypes. Single-cell RNA sequencing of murine asthmatic lung and myeloid-cell-specific *Tnfsf15*-knockout mice (*Tnfsf15*^Mac-KO^) was performed, and the effects of anti-TL1A interventions were evaluated in allergen-induced asthma models. **Results:** TL1A levels were significantly elevated in the serum and sputum of patients with asthma and correlated with clinical disease severity, declining lung function, and blood eosinophil counts. Single-cell RNA sequencing identified macrophages as the primary immune cells expressing TL1A in the context of allergic lung inflammation. In the *Tnfsf15*^Mac-KO^ mice, allergen-induced airway inflammation, T helper 2 cytokine secretion, and mucus hypersecretion were attenuated. Mechanistically, TL1A induced C-C motif chemokine ligand 8 (CCL8) expression via the activation of death receptor 3, thereby driving type 2 inflammation. Critically, the anti-TL1A antibody interventions suppressed T helper 2-mediated responses and reduced the number of pathogenic CD8^+^ T cells in a dose-dependent manner. **Conclusion:** TL1A serves a dual role as a biomarker and therapeutic target in asthma, as it modulates macrophage-driven pathogenesis. TL1A inhibition disrupts CCL8/C-C motif chemokine receptor 8 signaling and pathogenic T-cell responses, providing a precision medicine strategy for asthma.

## Introduction

Asthma is a serious health issue affecting over 300 million individuals worldwide, accounting for approximately 1% of the global disease burden [1]. Despite advances in maintenance therapies, the condition remains uncontrolled in a considerable proportion of patients with moderate to severe asthma, necessitating the development of novel therapeutic strategies [2]. In the era of personalized medicine, stratifying patients with asthma into different phenotypes and molecular endotypes to facilitate diagnosis and treatment remains a major challenge, which is accompanied by unmet clinical needs [3]. Further elucidating the mechanisms underlying asthma progression and identifying additional biological targets is vital for advancing precision treatment of asthma and achieving clinical remission [4].

Successful clinical research and drug trials have highlighted the importance of alarmins, which are being increasingly studied [5,6]. As major initiators of allergic immune responses, alarmins play an upstream role in the asthma-related inflammatory cascade and exhibit enormous potential for further exploration and applications in the treatment of a wider range of asthma phenotypes [6]. While identifying more effective potential therapeutic targets is important for improving asthma outcomes, it is equally crucial to develop a deeper understanding of the immune microenvironment in asthma and to ascertain the optimal timing for the administration of existing drugs [7].

In recent years, tumor necrosis factor-like ligand 1A (TL1A) was identified as a novel alarmin with great clinical potential. TL1A is encoded by the tumor necrosis factor superfamily member 15 (*TNFSF15*) gene on chromosome 9 in humans and the *Tnfsf15* gene on chromosome 4 in mice [8]. As a type II transmembrane protein, TL1A can exist in 2 forms, namely, a full-length membrane-bound form and a soluble form generated by matrix-metalloproteinase-mediated cleavage [9]. Unlike other members of the tumor necrosis factor (TNF) family, both forms of TL1A have been confirmed to be biologically active and functional, each of which exhibits the potential to exert differing or even opposing effects [9]. In terms of the cellular origin of the cytokines, studies have shown that TL1A can be produced by epithelial cells as well as macrophages, endothelial, and T cells [10], with its effects being mediated through various mechanisms, including cell–cell contact and direct stimulation [11]. Thus, the expression and cleavage of TL1A in source cells may change dynamically during disease progression [9,11]. In the context of asthma progression, previous studies have confirmed that TL1A plays a critical role in mediating eosinophilic airway inflammation and mucus hypersecretion [12,13]. Building upon earlier findings, several large international pharmaceutical companies have initiated clinical trials investigating interventions targeting TL1A [14–16]. Existing clinical trial data indicate that anti-TL1A monoclonal antibodies possess favorable pharmacokinetic and pharmacodynamic properties, along with acceptable safety and immunogenicity profiles, supporting their promising clinical potential [14–16].

Although TL1A functions as a pleiotropic cytokine in allergic airway environments, little is known about its expression dynamics and responsive cellular repertoire. Notably, beyond its known expression in epithelial cells, evidence suggests that macrophages may be one of the principal cell types producing TL1A in inflammatory settings. Our own studies and those of other groups have demonstrated that the cellular source of TL1A is a critical influencer of its functional impact and contributions to disease pathology [11,17,18]. Analysis of the LungMAP Human Lung Atlas has revealed constitutive TL1A expression in both alveolar epithelial and basal cells of healthy human lungs [19]. However, its expression pattern during allergic lung inflammation has yet to be definitively characterized—an important step toward understanding the underlying pathogenic mechanisms.

This study aimed to elucidate the mechanism by which TL1A modulates asthma development and to assess its utility as a biomarker. Associations were identified between elevated TL1A levels, asthma exacerbation, and the manifested phenotypes, with clinical severity correlating with the TL1A levels in the blood. Beyond its established expression in epithelial cells [18–20], this study identifies macrophages as a predominant and functionally critical TL1A-producing cell type in allergic lung inflammation, through the integration of single-cell RNA sequencing (scRNA-seq), flow cytometry, and cell sorting techniques. TL1A knockout (KO) or neutralization reduced interactions between macrophages and other immune cells while also inducing the most significant changes in gene expression, further confirming that macrophages are the primary sites of TL1A activity. Experiments integrating myeloid-cell-specific *Tnfsf15*-KO mice (*Tnfsf15*^Mac-KO^) and anti-TL1A neutralizing antibody interventions revealed the critical role of TL1A in orchestrating macrophage–C-C motif chemokine ligand 8 (CCL8)-mediated interactions and both T helper 2 (Th2)- and CD8 T-cell-driven pathology. Collectively, the findings suggest that TL1A has a dual role as both a biomarker and a therapeutic target, offering new avenues for precision asthma treatment.

## Results

### TL1A as the hallmark of severe asthma and an indicator of asthma control

**Fig. 1.**
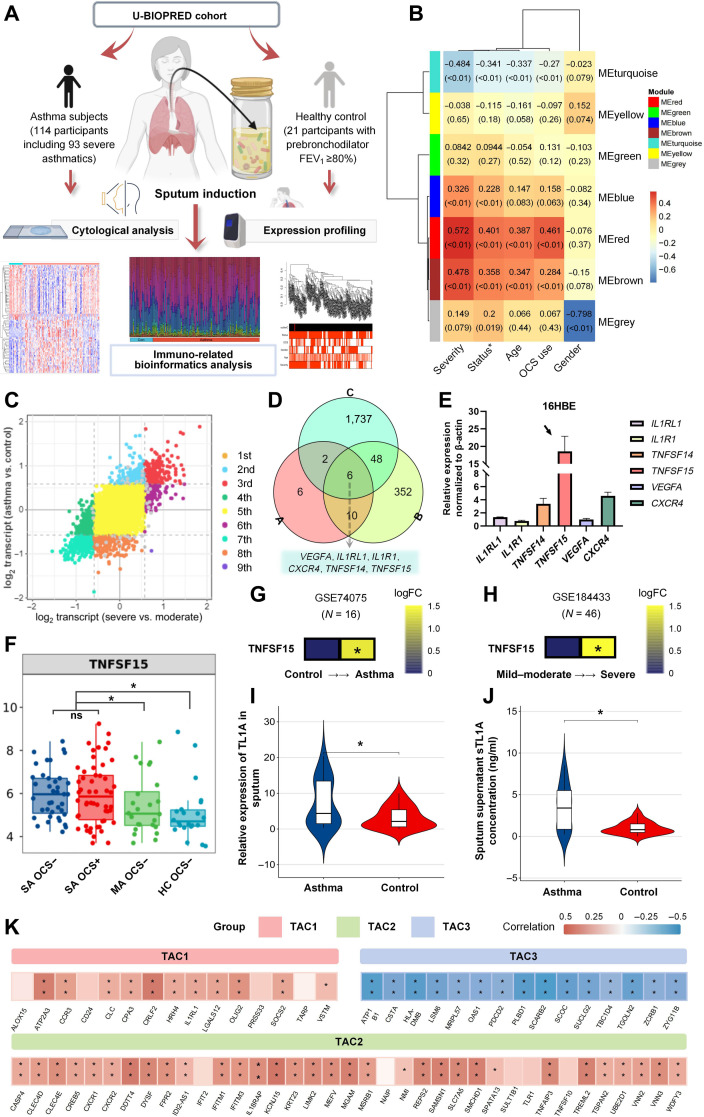
Tumor necrosis factor-like ligand 1A (TL1A) as a key immune marker in severe asthma. (A and B) Weighted coexpression network analysis (WGCNA) was conducted based on clinical characteristics and expression profiling data from the Unbiased Biomarkers for the Prediction of Respiratory Disease Outcomes (U-BIOPRED) cohort. The red and brown modules were identified as being significantly correlated with the clinical status of patients with asthma, including disease severity, the state of exacerbation, and oral corticosteroid usage. FEV_1_, forced expiratory volume in 1 s; OCS, oral corticosteroid. Status* refers to control status including the remission and acute exacerbation phases. (C) A 9-square grid plot was employed to screen differentially expressed genes (DEGs) associated with asthma severity (severe vs. mild asthma and asthma vs. healthy controls). (D) Venn diagram intersection analysis was performed to identify overlaps between the red/brown module genes, ImmPort immune-related gene sets, and the up-regulated DEGs linked to asthma severity. This analysis revealed 6 critical hub genes, namely, vascular endothelial growth factor A (*VEGFA*), interleukin 1 receptor-like 1 (*IL1RL1*), interleukin 1 receptor type I (*IL1R1*), C-X-C chemokine receptor 4 (*CXCR4*), tumor necrosis factor superfamily member 14 (*TNFSF14*), and tumor necrosis factor superfamily member 15 (*TNFSF15*). FC, fold change; GSE, gene set enrichment. (E) In vitro model of asthma exacerbation established through the co-administration of TNF-α and polyinosinic:polycytidylic acid (poly(I:C)) in human bronchial epithelial cells to explore the expression levels of 6 hub genes. (F) Box-and-whisker plots illustrating the expression levels of *TNFSF15* across asthma subgroups stratified by disease severity (SA, severe asthma; MA, mild asthma) and oral corticosteroid (OCS) treatment. Data are presented as mean ± standard deviation for intergroup comparisons, with statistical significance denoted by *P* < 0.05. (G and H) The relative expression of TL1A in the sputum of patients with asthma compared with that of normal controls was analyzed using Gene Expression Omnibus (GEO) datasets, including the GSE74075 and GSE184433 datasets. (I) Comparison of TL1A expression in the sputum of patients with asthma and healthy controls. (J) TL1A concentrations in sputum supernatants were measured using enzyme-linked immunosorbent assay (ELISA). (K) Association analysis of sputum transcriptome-associated cluster (TAC) gene expression signatures (TAC1: characterized by immune receptors interleukin 33 receptor, C-C chemokine receptor 3, and thymic stromal lymphopoietin receptor; TAC2, characterized by T helper 17 cells, neutrophil activation, and inflammasome activity; and TAC3a/b, characterized by genes involved in metabolic pathways, ubiquitination, and mitochondrial function) and the *TNFSF15* level in sputum. Data are presented as mean ± SD. For panels (I) and (J), *n* = 18 (asthma) and *n* = 18 (control) for sputum samples; for GEO analyses (G and H), sample sizes are indicated in the respective datasets. *P < 0.05 and **P < 0.01 compared with the respective control groups in (G) to (J). * and ** indicate a significant correlation in (K) (*P* < 0.05 or *P* < 0.01). Data analysis for experimental studies was performed in a blinded manner.

The Unbiased Biomarkers for the Prediction of Respiratory Disease Outcomes (U-BIOPRED) cohort is a representative asthma cohort characterized by both molecular and clinical heterogeneity. A weighted gene coexpression network analysis was conducted based on the induced sputum dataset obtained from the U-BIOPRED database, comprising 135 participants (114 patients with asthma and 21 healthy controls) (Fig. 1A). Seven coexpression modules were identified; among them, 2 key modules (red and brown) were significantly associated with asthma status (Fig. 1B). Nine-quadrant analysis led to the identification of genes whose differential messenger RNA expression patterns correlated with disease severity; these genes were predominantly localized within quadrants 3 and 7 (Fig. 1C). The intersectional analysis based on Venn diagrams constructed with the red/brown gene clusters, up-regulated differentially expressed genes associated with asthma severity, and ImmPort immune-related gene sets led to the identification of asthma-associated immunological targets, including *TNFSF15* (Fig. 1D). Co-administration of TNF-α with polyinosinic:polycytidylic acid (poly(I:C)) to model experimental asthma exacerbation led to a significant up-regulation of *TNFSF15* (the gene encoding TL1A) among 6 core response genes, suggesting its critical involvement in both acute asthma attacks and disease progression (Fig. 1E). Further analysis demonstrated that *TNFSF15* expression increased in a severity-dependent manner in patients with asthma and remained unaffected by oral glucocorticoid treatment (Fig. 1F), suggesting that it could represent a therapeutic target that allows for the bypassing of conventional anti-inflammatory mechanisms. Analysis of the public datasets confirmed that TL1A expression levels become up-regulated in the sputum of patients with asthma (Fig. 1G and H); this result was validated in the polymerase chain reaction (PCR)/enzyme-linked immunosorbent assay (ELISA) analyses in the present cohort (Fig. 1I and J). It should be noted, however, that TL1A was undetectable in 17 sputum samples, and 12 out of the 17 (70.6%) sputum samples were primarily from individuals with mild disease or healthy subjects. Despite this technical constraint that may overestimate true levels, particularly in milder cases, the measurable TL1A concentrations were significantly higher in the asthma group and correlated with disease severity among detectable samples (data not shown).

A recent study reported on the establishment of 3 well-characterized, asthma-associated, sputum transcriptome-associated clusters (TAC1, TAC2, and TAC3) [21]. Participants with the TAC3 cluster exhibited the lowest prevalence of severe asthma and oral corticosteroid dependency compared to participants with the other TAC clusters. Furthermore, TL1A expression exhibited a significant negative correlation with all TAC3-representative genes but was mainly positively correlated with TAC1/TAC2 (Fig. 1K).

Immune cell infiltration was also explored in patients with severe and moderate asthma and in healthy participants using the CIBERSORT algorithm (Fig. S1A). The CIBERSORT analysis revealed greater numbers of activated mast cells, dendritic cells, and eosinophils in the sputum of patients with asthma compared to the number in healthy controls (Fig. S1B). Dendritic cell activation and plasma cell differentiation were significantly enhanced in patients with severe asthma compared to that in patients with mild asthma (Fig. S1C). TL1A levels were positively correlated with the numbers of eosinophils (*r* = 0.38, *P* = 0.0015), activated mast cells (*r* = 0.42, *P* = 0.0021), plasma cells (*r* = 0.30, *P* = 0.0011), and dendritic cells (*r* = 0.51, *P* < 0.001) (Fig. S1D to G), underscoring the association of TL1A level with asthma severity.

### Elevated TL1A expression in the serum of patients with asthma and its relationship with the degree of asthma control

**Fig. 2.**
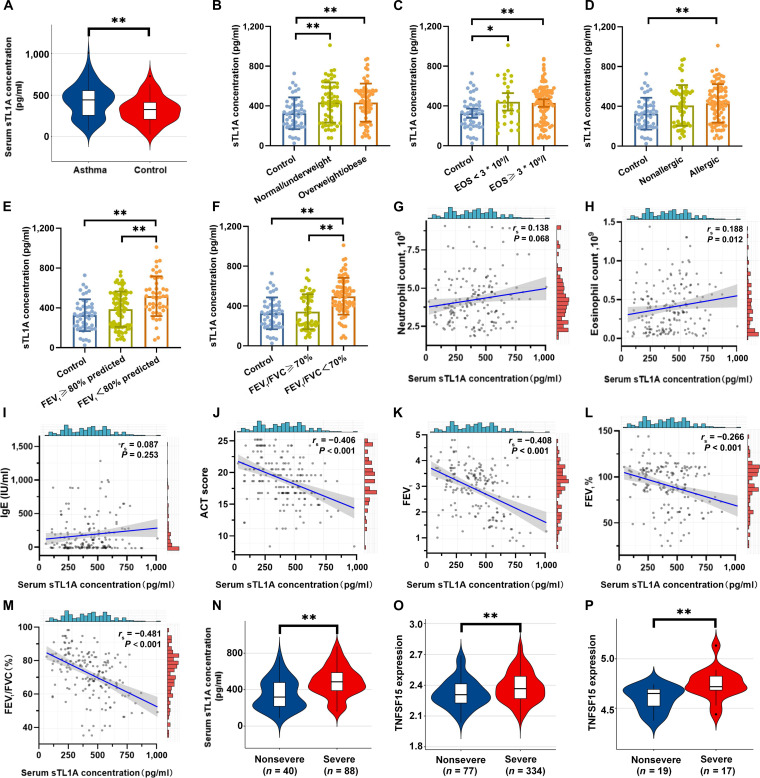
Assessment of relationships between tumor necrosis factor-like ligand 1A (TL1A) levels in serum and the clinical characteristics of patients with asthma. (A) Comparisons of serum concentrations of TL1A in the asthma and control groups. (B) Comparison of TL1A concentrations in various body mass index (BMI) categories among patients with asthma: normal or underweight (BMI < 24.0 kg/m^2^), overweight (BMI 24.0 to 27.9 kg/m^2^), and obese (BMI > 28.0 kg/m^2^). (C) Comparison of TL1A concentrations in patients with asthma with blood eosinophil (bEOS) concentrations of ≥300 and <300 cells/μl. (D) Comparison of TL1A concentrations between patients with allergic asthma and nonallergic asthma. (E) Comparison of TL1A concentrations in patients with asthma with prebronchodilator (pre-BD) forced expiratory volume in 1 s (FEV_1_) <80% predicted and pre-BD FEV_1_ ≥80% pred. (F) Comparison of TL1A concentrations in patients with asthma with pre-BD FEV_1_/forced vital capacity (FVC) <70% and pre-BD FEV_1_/FVC ≥70%. (G) Correlation analysis between neutrophil counts and TL1A concentrations. (H) Correlation analysis between eosinophil counts and TL1A concentration. (I) Correlation analysis between immunoglobulin E (IgE) levels and TL1A concentrations. (J) Correlation analysis between asthma control test (ACT) score and TL1A concentration. (K) Correlation analysis between FEV_1_ levels and TL1A concentrations. (L) Correlation analysis between FEV_1_% pred values and TL1A concentrations. (M). Correlation analysis between FEV_1_/FVC ratios and TL1A concentrations. (N) Comparisons of the serum concentrations of TL1A in the patients with severe asthma and patients with nonsevere asthma. (O and P) The level of TL1A expression in blood across asthma severity from public datasets (GSE69653 and GSE27011). Data are presented as mean ± SD. Serum samples: asthma group *n* = 128; control group *n* = 48 (A and N). For subgroup analyses (B to F), sample sizes are as indicated in the respective bars/scatter plots. Correlation analyses (G to M) include all asthma patients (*n* = 128). Public dataset analysis (O and P) sample sizes are as follows: GSE69653, *n* = 411, and GSE27011, *n* = 36. *P < 0.05 and **P < 0.01 compared with the respective control groups. Statistical analyses were performed blinded to group allocation.

Using our asthma biobank data, correlations were analyzed between serum TL1A levels and clinical features. No differences in baseline demographic variables were observed between the patients with asthma (*n* = 128) and the healthy controls (*n* = 48) (Table S1); however, the patients with asthma exhibited elevated systemic inflammatory markers (i.e., higher leukocyte, neutrophil, and eosinophil counts), higher serum levels of immunoglobulin E (IgE), and poorer lung function (Table S1). Serum TL1A expression was significantly elevated in patients with asthma compared to that in healthy controls (*P* < 0.01, Fig. 2A). While TL1A levels were higher across all asthma phenotypes compared to that in controls, they did not differ significantly by body mass index, blood eosinophil (bEOS) count (bEOS > 300 vs. bEOS ≤ 300 cells/μl), or allergic status (Fig. 2B to D). Critically, patients with severe airway obstruction exhibited higher TL1A levels (Fig. 2E and F), suggesting that it may play a role in mediating the development of treatment resistance. In the correlation analysis (Fig. 2G to M), serum TL1A levels were significantly correlated with bEOS counts (*r*_s_ = 0.188, *P* = 0.012) and asthma control test score (*r*_s_ = −0.406, *P* < 0.001). Furthermore, significant negative correlations were observed between TL1A levels and parameters of pulmonary function, including the forced expiratory volume in 1 s (FEV_1_) (*r*_s_ = −0.408, *P* < 0.001), the percentage of the predicted FEV_1_ (FEV_1_%) (*r*_s_ = −0.266, *P* < 0.001), and the FEV_1_/forced vital capacity ratio (*r*_s_ = −0.481, *P* < 0.001) (Fig. 2K to M).

Phenotypic characterization and endotyping are crucial in order to provide severe asthma population with potential personalized treatment. Subsequently, we moved to further explore the TL1A levels across asthma severities. In our data, serum TL1A expression was significantly elevated in severe asthma population compared to that in the nonsevere asthma group (*P* < 0.01, Fig. 2N). To further substantiate the aforementioned relationship, we explored 2 public datasets containing gene expression data from blood samples in the U-BIOPRED cohort (*n* = 411, including 334 patients with severe asthma and 77 with nonsevere asthma) and from pediatric asthma patients (*n* = 36, comprising 17 children with severe asthma and 19 with nonsevere asthma). For this analysis, blood samples from individuals with severe asthma exhibited an upward trend in TL1A expression (Fig. 2O and P), further supporting the relevance of TL1A as a biomarker in severe asthma.

### Asthma-progression-dependent elevation of TL1A expression in asthmatic mice

**Fig. 3.**
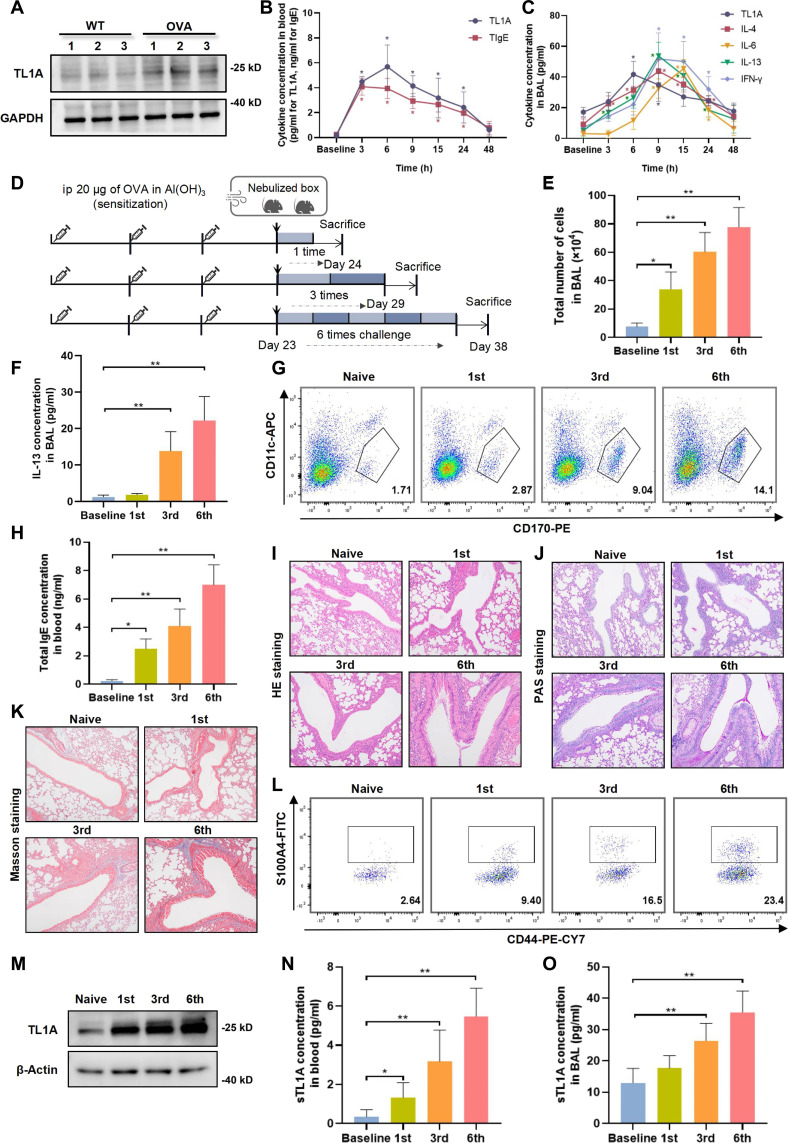
Tumor necrosis factor-like ligand 1A (TL1A) expression and secretion increase as asthma progresses. (A) Western blot analysis of TL1A protein expression in the lung tissues in the wild-type and ovalbumin (OVA)-induced model groups. (B) Changes in the cytokine concentrations of TL1A and immunoglobulin E (IgE) in serum over time following the final challenge in cases and controls. (C) Changes in the cytokine concentrations of TL1A, interleukin 4 (IL-4), interleukin 6 (IL-6), interleukin 13 (IL-13), and interferon gamma (IFN-γ) in bronchoalveolar lavage fluid (BAL) over time following the final challenge in cases and controls. (D) Schematic summarizing the timeline of the in vivo asthma modeling with different allergen exposure frequencies. (E) Comparison of total cell counts in the BAL in each group for different challenge numbers. (F) Comparison of IL-13 concentrations in BAL changed for different challenge numbers. (G) Representative plots showing changes in eosinophil counts for different challenge numbers. (H) Changes in IgE concentrations in blood samples for different challenge numbers. (I to K) Representative plots showing Masson staining, hematoxylin and eosin (HE) staining, and periodic acid–Schiff (PAS) staining of pulmonary airway tissue in mice for different challenge numbers. (L) Representative plots showing comparison of S100A4^+^CD8^+^ effector memory T (Tem) cell counts for different challenge numbers. (M) Comparison of TL1A expression level in lung tissue samples from each group. (N and O) Comparison of TL1A concentrations in serum/BAL samples in each group. Data are presented as mean ± SD (error bars) from at least 3 independent experiments, with *n* = 6 mice per group per experiment (A and E to O). Time-course studies (B and C) used *n* = 6 mice per time point. *P < 0.05 and **P < 0.01 compared with the respective control groups. Histological scoring and flow cytometry analysis were performed by investigators blinded to the experimental groups.

To further elucidate the role of TL1A in asthma and its correlation with disease severity, allergen-induced asthma models were established in mice, including an acute model and a high-severity model involving prolonged allergen exposure. TL1A protein expression was significantly increased in the lung tissue of asthmatic mice induced by ovalbumin (OVA) or house dust mite (HDM) compared to that in control mice (Fig. 3A and Fig. S2). Following allergen challenge, the serum TL1A levels increased within 3 h and peaked at 6 h, whereas IgE declined after 3 h (Fig. 3B). The TL1A levels in bronchoalveolar lavage fluid (BAL) samples peaked earlier than the Th2-derived cytokines did (for interleukin 4 [IL-4]/interleukin 13 [IL-13]), confirming its function as an alarmin (Fig. 3C). Given the established links between prolonged allergen exposure and asthma severity [22], models involving incremental allergen challenges were established (0, 1, 3, and 6 exposures, with sacrifice performed 24 h after the final challenge; Fig. 3D). Inflammatory cell recruitment in the airway is a characteristic feature of the pathophysiology of asthma. In the present study, the number of cells in the BAL samples increased markedly as the allergen exposure frequency increased (Fig. 3E). Furthermore, the IL-13 levels in the BAL samples (Fig. 3F), the number of eosinophils in lung tissue (gated on CD170^+^CD11c^−^ in Fig. 3G), and the serum IgE levels (Fig. 3H) increased in accordance with the allergen stimulation times. The histological analysis revealed exposure-dependent increases in the degree of peribronchial inflammation, goblet cell hyperplasia, and collagen deposition (Fig. 3I to K). There was also an increase in the number of CD8^+^S100A4^+^ effector memory T (Tem) cells, a known pathogenic subset of CD8^+^ Tem cells, which corresponded with the frequency of allergen exposure, consistent with the results of a previous observational study [22]. Critically, the TL1A levels in serum, BAL, and lung homogenates increased progressively in accordance with both the exposure frequency and pathological severity (Fig. 3M to O), indicating that TL1A has potential utility as a biomarker and may be a contributor to asthma pathogenesis.

### Role of macrophages in TL1A-induced allergic airway inflammation

**Fig. 4.**
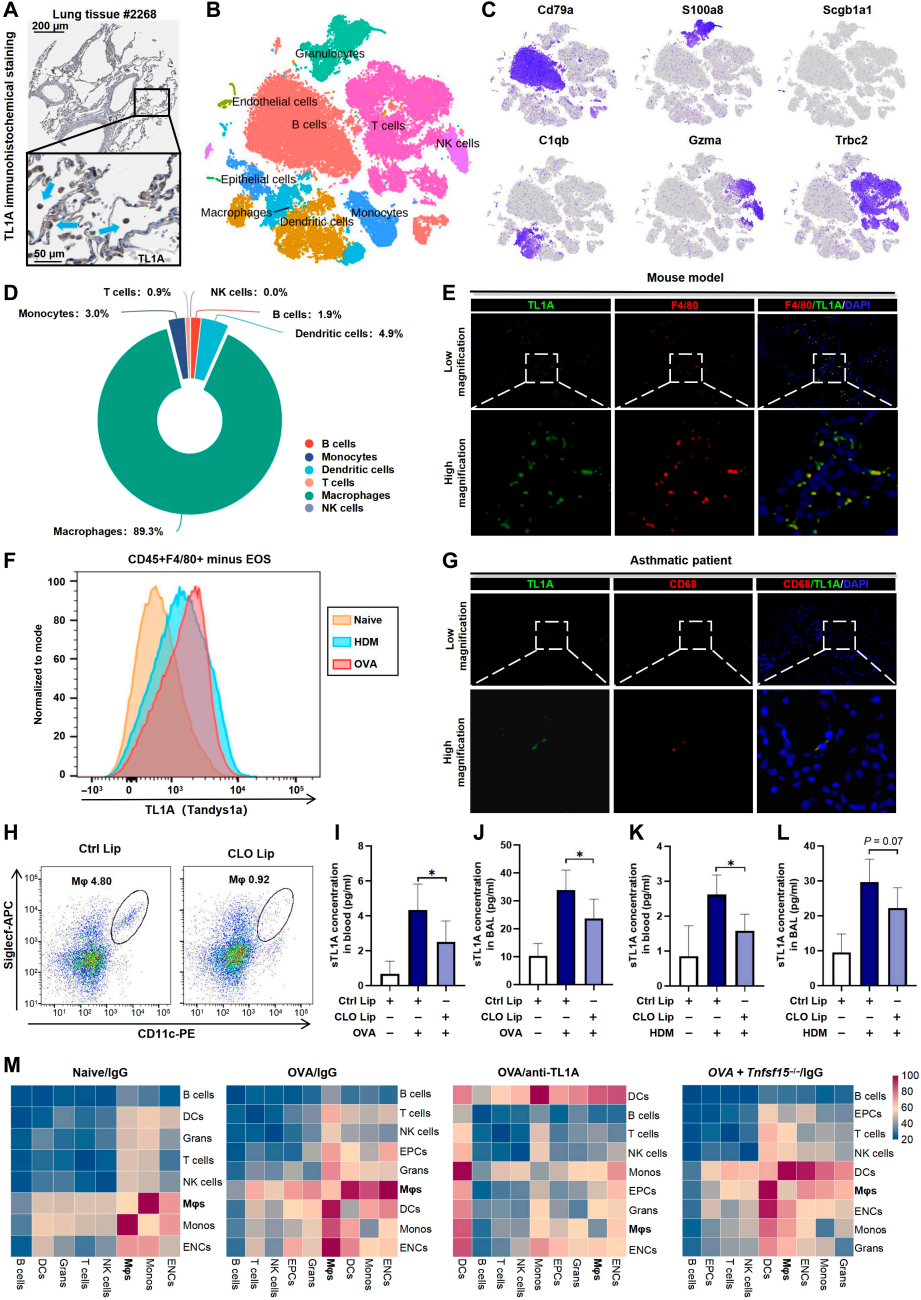
Identification and verification of the major tumor necrosis factor-like ligand 1A (TL1A)-expressing cell populations in the context of allergic lung inflammation. (A) Results of the immunohistochemical staining of TL1A in human lung tissue from the Human Protein Atlas (HPA) database. (B) Single-cell profiling via t-distributed stochastic neighbor embedding (t-SNE) following initial annotation using SingleR. (C) Cell clustering and selections made according to manual curation for the degree of relevance based on a marker gene. (D) Pie chart showing the TL1A expression distribution based on single-cell RNA sequencing (scRNA-seq). NK, natural killer. (E) Sample images of the colocalization of macrophages with TL1A in the lung tissue of asthmatic model mice. (F) Representative plots showing the number of TL1A^+^ macrophages (CD45^+^F4/80^+^ cells minus eosinophils). (G) Sample images of the colocalization of human macrophages with TL1A in the airways of patients with asthma. (H) Representative plots revealing the depletion of CD170^+^CD11c^+^ macrophages in lung tissue after treatment with clodronate (CLO) liposomes. (I to L) Changes in TL1A concentrations in serum/bronchoalveolar lavage fluid (BAL) after CLO liposome treatment. (M) Cell–cell communications between macrophages and other cell types were substantially altered after administration of an anti-TL1A antibody or *Tnfsf15* depletion in the mouse model. DCs, dendritic cells; ENCs, endothelial cells; EPCs, epithelial cells; Grans, granulocytes; Monos, monocytes; Mφs, macrophages. Data are presented as mean ± SD. scRNA-seq analysis (B to D) was performed on lung cells pooled from 4 mice. Flow cytometry and imaging data (E and F) are representative of 3 independent experiments with *n* = 3 mice per group. For CLO experiments (H to L), *n* = 6 mice per group. All quantifications were performed in a blinded fashion.

Single-cell studies have identified alveolar epithelial and airway basal cells as primary sources of constitutive TL1A expression in healthy human lungs [18]. However, under inflammatory conditions, TL1A is predominantly recognized as an inducible cytokine secreted by immune and endothelial cells [11]. As the cellular origin is a critical determinant of the pathogenic impact of TL1A, confirming the dominant TL1A-producing cells during allergic airway inflammation was key motivation in this study. Human Protein Atlas analysis revealed pulmonary macrophages as the major source of TL1A alongside epithelial cells (Fig. 4A). The scRNA-seq analysis of the OVA-induced asthmatic murine lung tissue identified 9 cell clusters, with TL1A being predominantly localized within macrophages (Fig. 4B to D), which aligned with the results of analyses based on LungMAP data [19]. Consistent with previous findings, in addition to being observed to localize in the airway lumen (primarily in epithelium), TL1A also partially colocalized with F4/80 (Fig. 4E). The flow cytometric analysis further validated the elevation of TL1A levels in CD45^+^F4/80^+^ cells (Fig. 4F). Parallel observations confirmed the colocalization of TL1A–CD68 in biopsies of asthmatic tissue in humans (Fig. 4G). Clodronate depletion eliminated 80% of the CD170^+^CD11c^+^ macrophages (Fig. 4H), thereby reducing TL1A levels in OVA-induced models to 60% (in serum) and 75% (in BAL) (Fig. 4I and J). Results from the HDM-induced models were also similar (Fig. 4K and L). These results confirmed that macrophages are the principal cellular source of TL1A. Although technical limitations precluded the identification of discrete macrophage subsets with high TL1A expression, the cell–cell communication analysis following TL1A KO/neutralization revealed a marked reduction in macrophage-mediated signaling, with macrophage interactions yielding the most differentially expressed genes (Fig. 4M). Thus, macrophages may not only produce TL1A but also mediate TL1A-dependent allergic responses.

### Attenuation of allergic airway inflammation via KO of myeloid lineage-specific TL1A

**Fig. 5.**
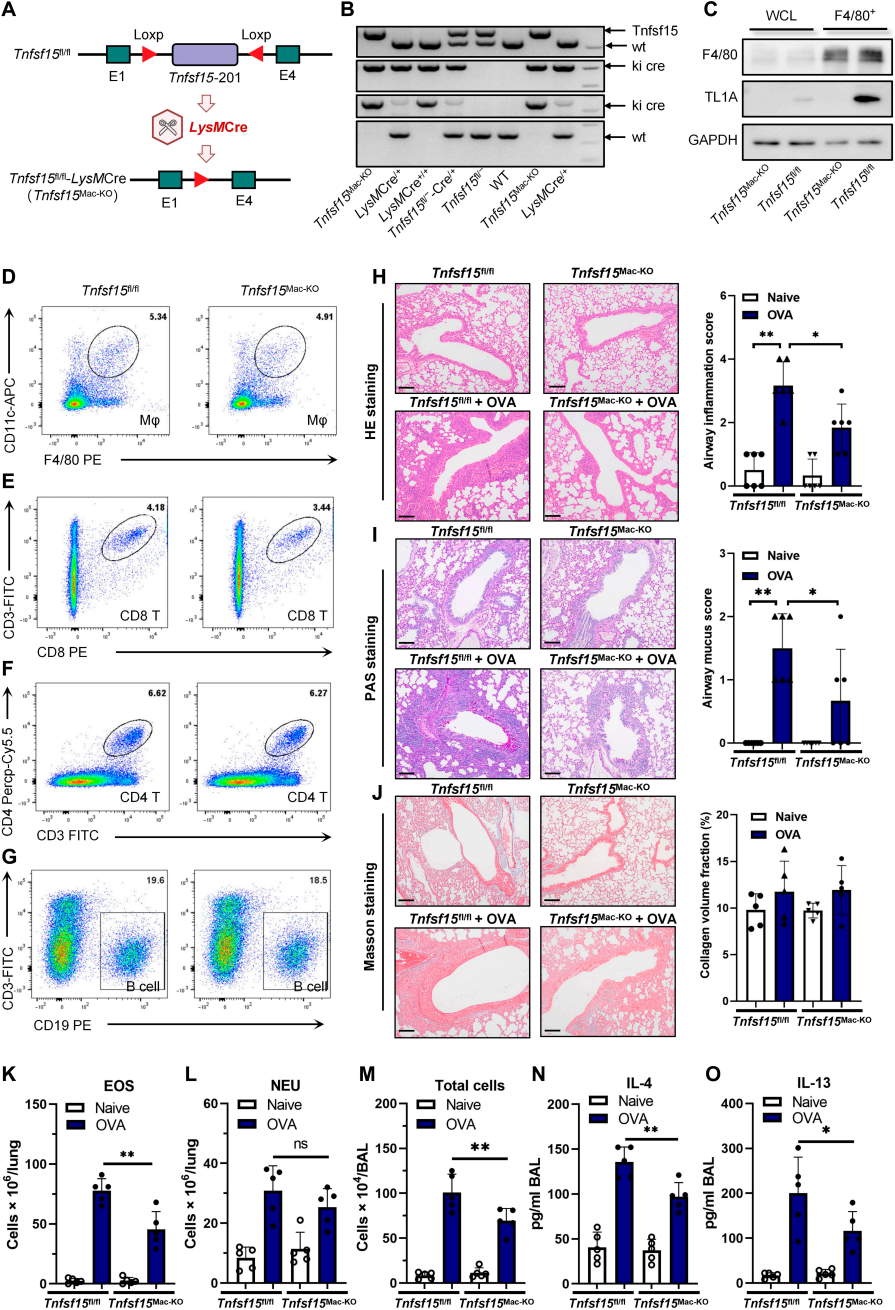
Myeloid-cell-specific *Tnfsf15*-knockout resulted in the attenuation of allergic airway inflammation. (A) Myeloid-cell-specific *Tnfsf15*-knockout strategy. (B) Genotyping was confirmed using tail DNA genomic polymerase chain reaction. (C) Immunomagnetic bead sorting was used to isolate F4/80^+^ cells, and the expression level of tumor necrosis factor-like ligand 1A (TL1A) was quantified using Western blotting. (D to G) Representative plots showing the proportions of 4 major types of immune cells (macrophages, CD8^+^ T cells, CD4^+^ T cells, and B cells) infiltrating the lung tissues in each group. (H) Representative hematoxylin and eosin (HE) staining among the different groups and quantification of the airway inflammation score. Black scale bar, 50 µm. (I) Representative periodic acid–Schiff (PAS) staining among the different groups and quantification of the airway mucus score. (J) Representative Masson’s trichrome staining among the different groups and quantification of the collagen volume fraction. (K and L) Lung eosinophil or neutrophil counts, as determined by flow cytometry. (M) Total cell counts in bronchoalveolar lavage fluid (BAL). (N) Concentrations of interleukin 4 (IL-4) in BAL. (O) Concentrations of interleukin 13 (IL-13) in BAL. Data are presented as mean ± SD. Experiments were repeated 3 times with *n* = 6 to 8 mice per group per experiment. Cell sorting and Western blot (C) used pooled cells from *n* = 4 mice. Flow cytometry analyses (D to G and K) and histological scoring (H to J) were performed by investigators blinded to genotype and treatment. *P < 0.05 and **P < 0.01 compared with the respective groups.

To determine whether TL1A-producing macrophages are essential for the development of asthma, myeloid lineage-specific TL1A KO mice (*Tnfsf15*^Mac-KO^) were generated by crossing *Tnfsf15*^fl/fl^ mice with *Lyz2*-Cre mice, and the genotype was confirmed using PCR (Fig. 5A and B). F4/80^+^ cells were isolated to evaluate the KO efficiency in vivo. The F4/80^+^ cells sorted from the *Tnfsf15*^Mac-KO^ mice exhibited a significant reduction in TL1A protein levels compared to those in the controls (Fig. 5C). Flow cytometry revealed no abnormalities in the major immune cell populations (macrophages, CD4^+^ T cells, CD8^+^ T cells, and B cells) (Fig. 5D to G), excluding developmental defects in the *Tnfsf15*^Mac-KO^ mice (Fig. 5D to G). The histology revealed a reduction in both peribronchial inflammation and goblet cell metaplasia in the allergen-treated *Tnfsf15*^Mac-KO^ mice (Fig. 5H and I and Fig. S3A and B), although collagen deposition was unaffected (Fig. 5J). Similarly, the allergen-treated *Tnfsf15*^Mac-KO^ mice exhibited considerably decreased infiltration of eosinophils (Fig. 5K and Fig. S3C) in the lungs, diminished total cell counts (Fig. 5L and Fig. S3E), and lower expression levels of IL-4 and IL-13 (Fig. 5M and N and Fig. S3F and G) in the BAL samples compared with those in the allergen-treated *Tnfsf15*^fl/fl^ mice. Collectively, these results indicated that Th2 cells responses decreased in the *Tnfsf15*^Mac-KO^ mice.

Compared to the OVA-induced model, the HDM-induced murine model of asthma exhibited more prominent neutrophil infiltration; this phenomenon was partially reversed in *Tnfsf15*^Mac-KO^ mice (Fig. S3D). Further investigation revealed that HDM-induced asthmatic responses were attenuated upon myeloid-cell-specific *Tnfsf15* KO, which was associated with a diminished T helper 1 (Th1) inflammatory response (Fig. S3H). Concurrently, decreased levels of multiple neutrophil-associated chemokines were observed (Fig. S3I and J), collectively suggesting that TL1A may modulate the progression of asthmatic airway inflammation through multiple pathways.

### Induction of CCL8 by TL1A and its function as an amplifier of allergic inflammation

**Fig. 6.**
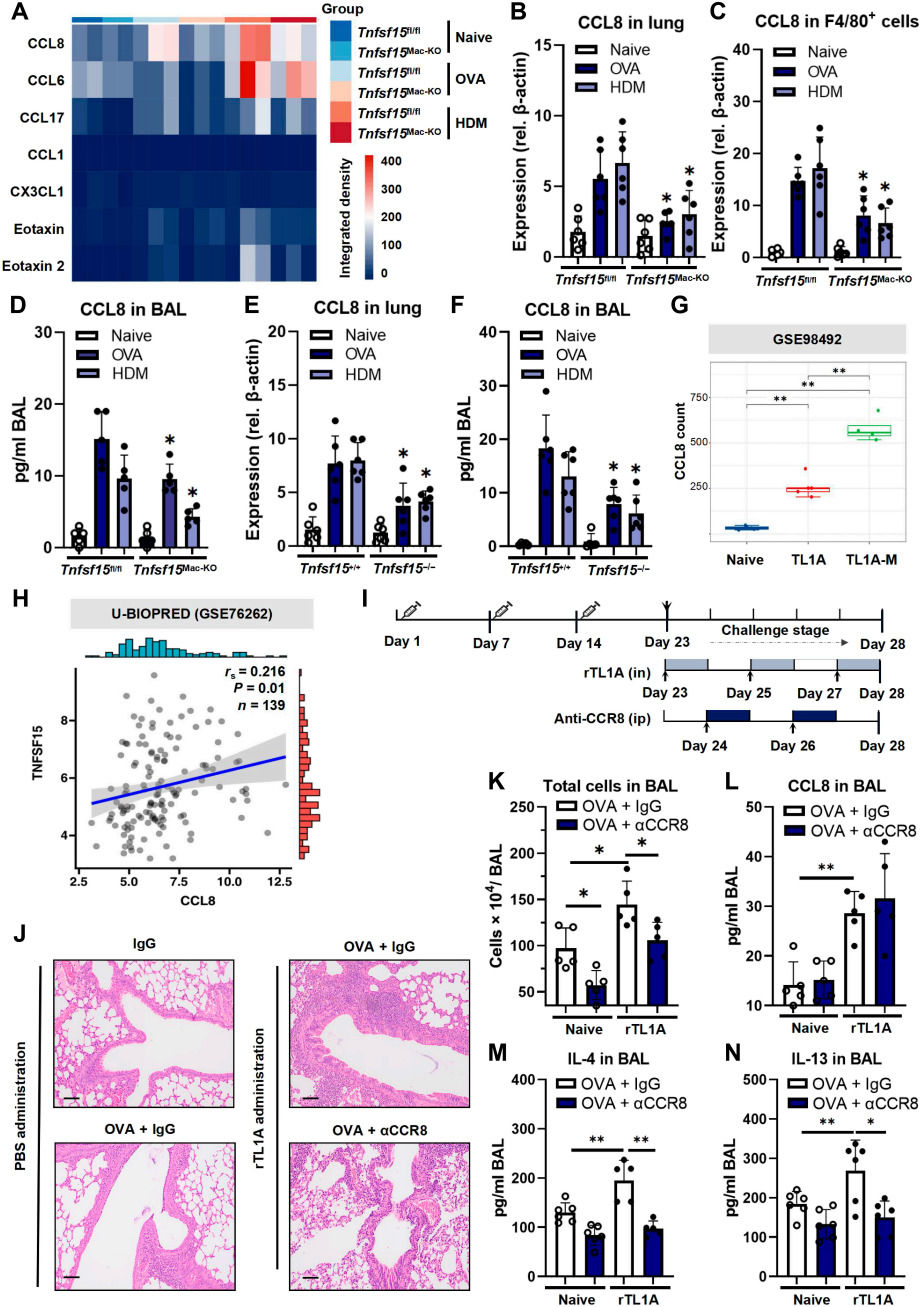
Identification of C-C motif chemokine ligand 8 (CCL8) as the key link in the tumor necrosis factor-like ligand 1A (TL1A)-induced allergic inflammatory response. (A) Heatmap showing changes in proallergic chemokine expression levels after gene knockdown. (B) Relative gene expression of CCL8 in lung tissue, quantified using polymerase chain reaction (PCR). (C) Relative gene expression of CCL8 in sorted F4/80^+^ cell groups in lung tissue, quantified using PCR. (D) CCL8 concentration in bronchoalveolar lavage fluid (BAL) measured using enzyme-linked immunosorbent assay (ELISA). (E) Relative gene expression of CCL8 in lung tissue, quantified using PCR in *Tnfsf15*-knockout (KO) mice. (F) CCL8 concentration in BAL measured using ELISA in *Tnfsf15*-KO mice. (G) CCL8 expression in the lung tissue of *Tnfsf15* transgenic and nontransgenic mice. (H) The correlation between TL1A and CCL8 expression in the Unbiased Biomarkers for the Prediction of Respiratory Disease Outcomes (U-BIOPRED) cohort (based on GSE76262). (I) Schematic of the murine ovalbumin-induced asthma models following recombinant TL1A or anti-C-C motif chemokine receptor 8 (anti-CCR8) intervention. (J) Hematoxylin and eosin (HE) staining of lung tissue after allergen challenge and recombinant TL1A (rTL1A) or anti-CCR8 interventions. Black scale bar, 50 µm. (K) Total cell numbers in BAL samples. (L to N) Concentrations of CCL8, interleukin 4 (IL-4), and interleukin 13 (IL-13) in BAL samples from the different groups. Data are presented as mean ± SD from at least 2 independent experiments, with *n* = 5 to 6 mice per group per experiment. PCR, ELISA, and histological assessments were conducted by researchers blinded to treatment groups. Public dataset correlation (H) used *n* = samples from the GSE76262 dataset. *P < 0.05 and **P < 0.01 compared with the respective groups.

Given the pivotal role of macrophages in mediating type 2 immune responses to allergens [23], it was hypothesized that the attenuated inflammation observed in *Tnfsf15*^Mac-KO^ mice stemmed from the genetic deletion of TL1A and the resulting impairment of macrophage function. It is well-known that macrophages predominantly mediate asthma through both their secretion of proallergic cytokines and their participation in antigen presentation [24–26]. Bulk RNA sequencing (RNA-seq) screening of allergen-challenged lung tissues revealed CCL8, a macrophage-derived chemokine that amplifies type 2 inflammation, as the most substantially down-regulated proallergic factor (Fig. 6A) [26–29]. This was confirmed in the lung tissue of *Tnfsf15*^Mac-KO^ mice, in F4/80^+^ cells using reverse transcription PCR (RT-PCR), and in BAL using ELISA (Fig. 6B to D). Importantly, the reduced expression of CCL8 was recapitulated in conventional TL1A KO (TL1A^−/−^) tissue and BAL (Fig. 6E and F), whereas the analysis of the Gene Expression Omnibus data revealed up-regulated expression of CCL8 in TL1A-transgenic lungs (Fig. 6G). The clinical relevance of these findings was confirmed in the U-BIOPRED cohort analysis, which revealed a significant correlation between *TNFSF15* and *CCL8* expression in asthmatic sputum (*r*_s_ = 0.216, *P* = 0.01; Fig. 6H).

To establish a direct link, recombinant TL1A (rTL1A) was administered to the 2 independent asthmatic model mice (OVA or HDM, Fig. 6I to N and Fig. S4). Both models showed that the expression of CCL8 improved with TL1A treatment. The TL1A-treated mice exhibited increased CCL8 expression accompanied by aggravated lung inflammation, as evidenced by the increases in peri-airway inflammatory aggregates (Fig. 6J and Fig. S4A), total cell numbers in BAL samples, and Th2 cytokine levels (Fig. 6K to N and Fig. S4B to E).

C-C motif chemokine receptor 8 (CCR8) has been reported to be the primary functional receptor for CCL8 in allergic inflammation [26,28,29]. scRNA-seq analysis revealed that CCR8 was predominantly expressed in T cells (Fig. S5A). Further subset analysis identified enriched CCR8 expression specifically in asthma-relevant populations (notably Th2 and regulatory T [Treg] cells), suggesting a potential important role for the CCL8–CCR8 axis in asthma pathogenesis (Fig. S5B). We also measured CCL1, an alternative ligand for CCR8. Consistent with previous reports [26], under allergic inflammation conditions, CCL1 expression was substantially lower than that of CCL8 (Fig. 6A), supporting the premise that CCR8-mediated effects in this context are predominantly driven by CCL8. Next, we used a CCR8-blocking antibody to intervene in mice treated with rTL1A, aiming to dissect the dependency of rTL1A-induced effects on CCL8–CCR8 signaling. As shown, CCR8 blockade reversed the rTL1A-induced pathology without altering CCL8 levels (Fig. 6J to N and Fig. S4), indicating that CCL8 partially mediates TL1A-driven inflammation via CCR8.

### Mechanism of TL1A–CCL8 axis activation in vitro

**Fig. 7.**
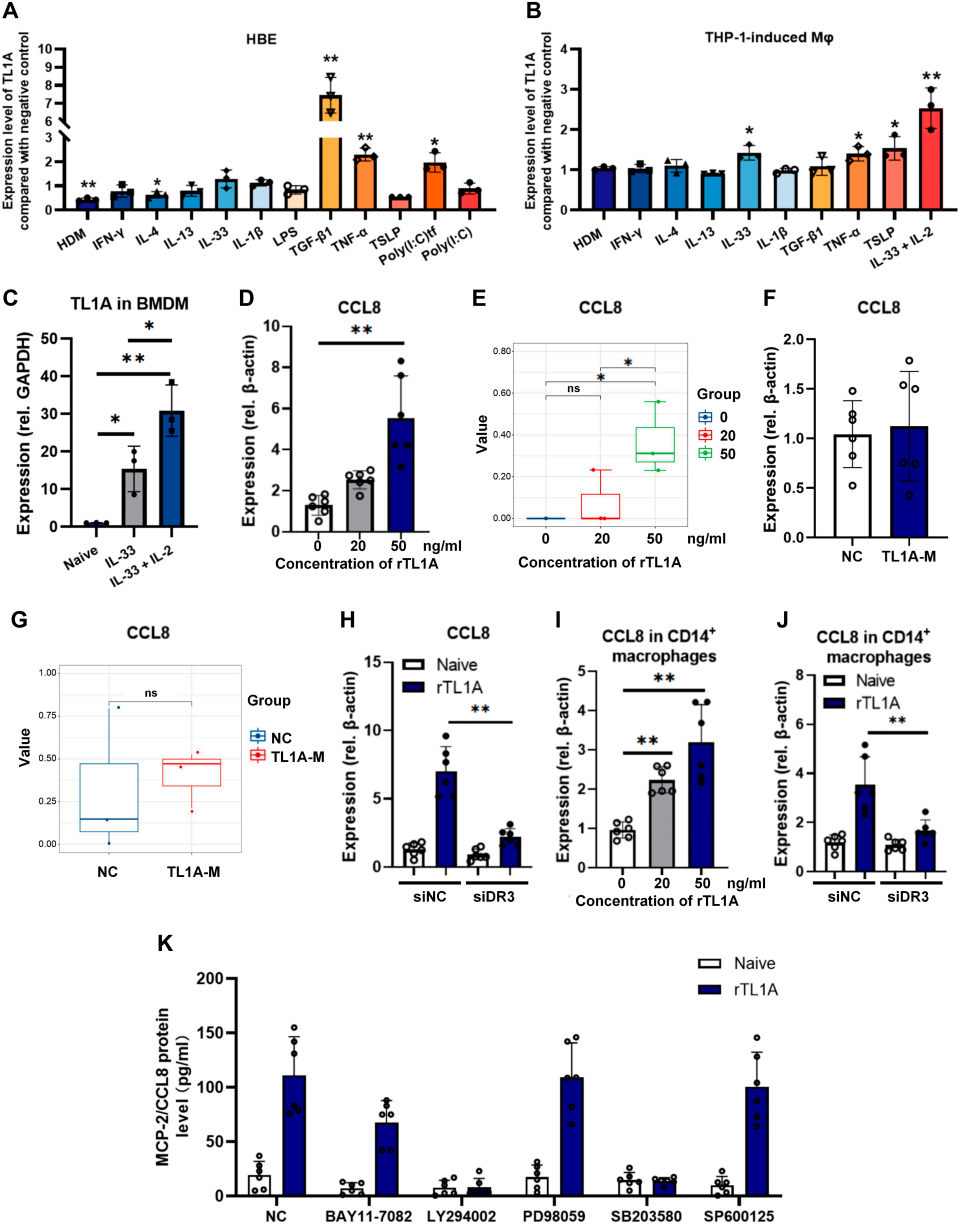
Mechanism underlying tumor necrosis factor-like ligand 1A (TL1A)–C-C motif chemokine ligand 8 (CCL8) axis activation in the context of allergic inflammation. (A and B) Expression levels of TL1A induced by different stimuli in human airway epithelial cells or macrophages. (C) TL1A expression in uninduced macrophages, macrophages induced by interleukin 33 (IL-33), and macrophages induced by IL-33 plus interleukin 2 (IL-2). (D and E) Comparison of the expression levels of CCL8 induced by different concentrations of TL1A, quantified using reverse transcription polymerase chain reaction (RT-PCR) or transcriptomic sequencing. The messenger RNA (mRNA) levels of CCL8 after the transfection of TL1A-M plasmids, quantified using RT-PCR (F) or transcriptomic sequencing (G). (H) Death receptor 3 (DR3) knockdown abolished the elevation of CCL8 induced by TL1A stimulation. (I) CCL8 level in CD14^+^ macrophages treated or not with TL1A. (J) RT-PCR analysis of CCL8 transcript following DR3 small interfering RNA (siRNA) treatment. Representative data from CD14^+^ cells from a single donor. (K) A schematic for the administration of different signaling pathway inhibitors to investigate the pathways involved in TL1A–CCL8 axis activation (BAY11-7082, a nuclear factor kappa B [NF-κB] inhibitor; LY294002, a phosphoinositide 3-kinase [PI3K inhibitor]; PD98059, an extracellular signal-regulated kinase [ERK] inhibitor; SB203580, a p38 inhibitor; and SP600125, a c-Jun N-terminal kinase [JNK] inhibitor). Data are presented as the mean ± SD of triplicate measurements from at least 3 independent cell culture experiments. For primary human macrophage experiments (I and J), cells were derived from *n* = 4 healthy donors. *P < 0.05 and **P < 0.01 compared with the respective groups. All in vitro stimulation and inhibitor treatments were analyzed in a blinded manner.

Although TL1A functions as an epithelial alarmin, its regulatory mechanism in airway epithelium remains unclear. Therefore, in vitro models were employed to elucidate the molecular mechanisms underlying the activation of the TL1A–CCL8 axis. Using human bronchial epithelial (HBE) cells, transforming growth factor beta (TGF-β) and TNF-α were confirmed to be potent TL1A inducers (Fig. 7A) [30,31]. Notably, transfection with poly(I:C) (simulating viral RNA via retinoic acid-inducible gene 1 [RIG-I]/melanoma differentiation-associated protein 5 [MDA5] activation) resulted in the robust up-regulation of TL1A levels, whereas direct poly(I:C) stimulation (toll-like receptor 3 [TLR3] mediated) induced minimal effects, highlighting the significance of TL1A in viral asthma exacerbation. In macrophages, exposure to alarmins (interleukin 33 [IL-33] and thymic stromal lymphopoietin [TSLP]) significantly up-regulated TL1A expression (Fig. 7B), contrasting with epithelial responses (Fig. 7B). Given the critical role of IL-33/interleukin 2 (IL-2)-induced modulation of macrophage states in allergic inflammation [32], macrophages were costimulated with IL-33/IL-2, which resulted in a pronounced regulatory effect on TL1A expression (Fig. 7B). These findings were corroborated in murine bone-marrow-derived macrophages (BMDMs) (Fig. 7C). These results demonstrated the context-dependent regulation of TL1A by immunological cues.

TL1A-mediated CCL8 induction was subsequently investigated in macrophages in vitro. Given the distinct functions of the soluble (sTL1A) and membrane-bound (mTL1A) forms of TL1A [9,17], macrophages were either treated with recombinant sTL1A or transfected with mTL1A-expression plasmids. Consistent with the RNA-seq data, the RT-PCR analysis confirmed that sTL1A induced a dose-dependent up-regulation of CCL8 (Fig. 7D and E), whereas mTL1A overexpression had no effect (Fig. 7F and G). Death receptor 3 (DR3) knockdown abolished the sTL1A-induced up-regulation of CCL8 (Fig. 7H), consistent with the broad DR3 expression patterns reported in macrophages across various pathologies [10,33]. As in THP-1 macrophages, in primary human monocyte-derived macrophages, TL1A stimulation increases CCL8 expression; small interfering RNA silencing of DR3 was associated with reduced CCL8 expression (Fig. 7I and J). Collectively, these results suggest that soluble ligand–receptor interactions may play a role in TL1A–CCL8 signaling in macrophages, although further in vivo validation is needed to clarify the physiological relevance of these distinct TL1A forms.

Previous studies have shown that extracellular TL1A engages multiple signaling pathways, including those involving nuclear factor kappa B (NF-κB), mitogen-activated protein kinase (MAPK), and p38, to modulate downstream effector responses [34,35]. The pharmacological inhibition experiments revealed that sTL1A activates CCL8 through NF-κB (BAY11-7082), phosphoinositide 3-kinase (PI3K) (LY294002), and p38 (SB203580), but not extracellular signal-regulated kinase (ERK) (PD98059) or c-Jun N-terminal kinase (JNK) (SP600125) pathways (Fig. 7I). These findings were indicative of synergistic cross talk between NF-κB, PI3K, and p38 signaling cascades in the TL1A-driven production pf CCL8, thereby amplifying allergic inflammation.

### Targeting TL1A as an efficient treatment strategy for asthma

**Fig. 8.**
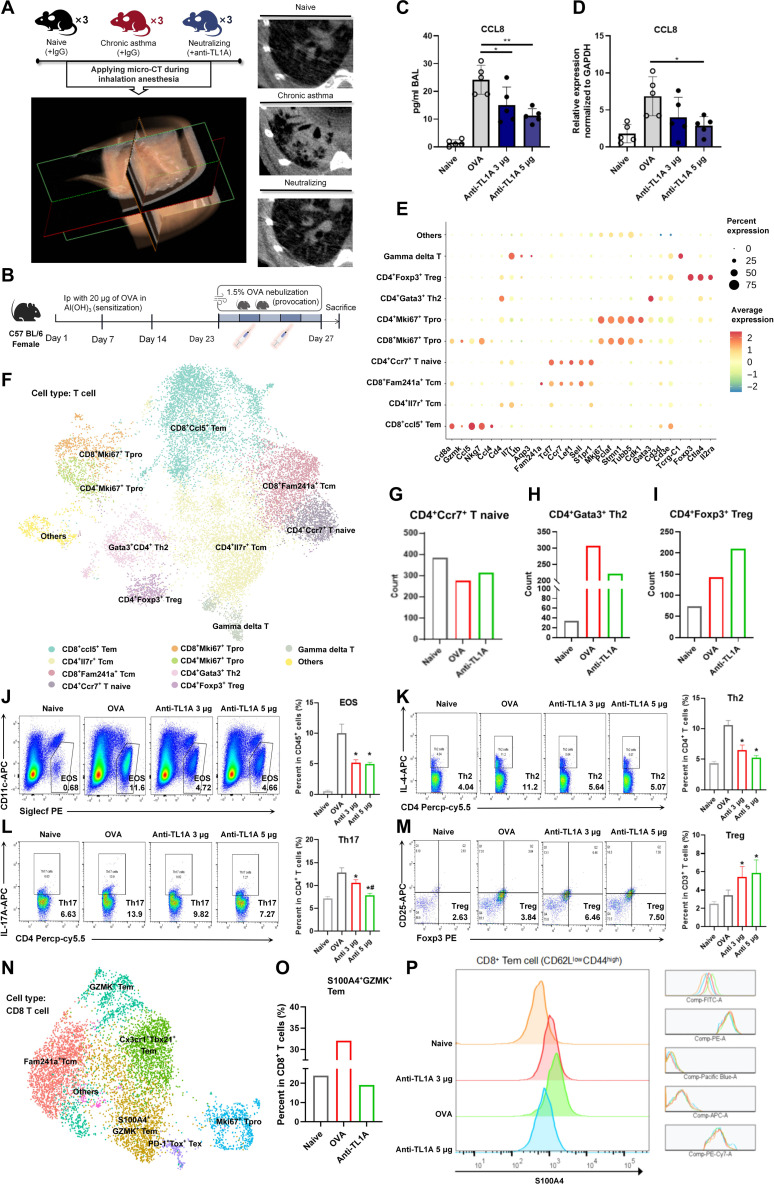
Targeted therapy based on anti-tumor necrosis factor-like ligand 1A (anti-TL1A) interventions in vivo. (A) Representative micro-computed tomography (micro-CT) images in each group. (B) Experimental schematic for the anti-TL1A interventions in the acute asthma model. (C and D) Effects of the anti-TL1A intervention on C-C motif chemokine ligand 8 (CCL8) expression in serum and lung tissue in the different experimental groups. (E and F) Partitioning diagram for the subsets of T cells identified through single-cell sequencing based on marker genes. (G to I) Proportional distribution characteristics of the main T-cell subsets (naive T cells, T helper 2 [Th2] cells, and regulatory T [Treg] cells) in single-cell samples. (J) The percentages of eosinophils were analyzed by flow cytometry. (K) The percentages of Th2 cells were analyzed by flow cytometry. (L) The percentages of T helper 17 (Th17) cells were analyzed by flow cytometry. (M) The percentages of Treg cells were analyzed by flow cytometry. (N) Results of the CD8^+^ T-cell refinement analysis. (O) Proportions of S100A4^+^GZMK^+^CD8^+^ effector memory T (Tem) cells in CD8^+^ T cells. GZMK, granzyme K. (P) Representative plots showing S100A4 intracellular staining of CD8^+^ Tem cells (CD62L^low^CD44^high^). Data are presented as mean ± SD. In vivo intervention studies used *n* = 6 mice per group, repeated twice. Single-cell RNA sequencing (scRNA-seq) analysis (E to I, N, and O) was performed on T cells isolated from *n* = 4 mice pooled for sequencing. *P < 0.05 and **P < 0.01 compared with the respective groups; ^#^*P* < 0.05, compared with the anti-TL1A 3-μg treatment group. Flow cytometry analyses (J to M and P) and micro-CT quantification were conducted by blinded investigators.

Few studies have investigated the effects of anti-TL1A antibody administration in asthma [36]; therefore, the therapeutic potential of the intervention was initially evaluated using micro-computed tomography (micro-CT) imaging to quantify pathological changes in vivo in a high-severity model involving prolonged allergen exposure. The anti-TL1A intervention markedly attenuated the pathological changes observed in the model mice (Fig. 8A). The scRNA-seq revealed that T lymphocytes were the primary TL1A targets via DR3 (Fig. S6A), which is consistent with known DR3 expression patterns. In the OVA-challenged mice (Fig. 8B), the anti-TL1A intervention resulted in a dose-dependent reduction in CCL8 expression (Fig. 8C and D). T-cell clustering led to the identification of naive, memory, effector, proliferative, regulatory, and γδ subsets (Fig. 8E and F). Allergen exposure decreased the number of naive T cells while simultaneously increasing the number of GATA binding protein 3-positive (GATA3^+^) CD4^+^ Th2 cells (Fig. 8G and H). Anti-TL1A treatment reversed this imbalance, reducing the number of Th2 cells and expanding the population of Foxp3^+^ Treg cells (Fig. 8H and I). As shown in Fig. 8J, the number of eosinophils expressing CD170 was significantly increased in the group treated with OVA alone; however, after anti-TL1A intervention, this upward trend was reversed. The flow cytometry also revealed a decreased percentage of IL-4^+^ and IL-17A^+^CD4^+^ T cells and an increased percentage of Foxp3^+^CD4^+^ T cells in the anti-TL1A group (Fig. 8K to M).

The anti-TL1A intervention also modulated CD8^+^ T-cell compartments (Fig. 8I and Fig. S6B). Focusing on the pathogenic CD8^+^ subsets of Tem cells that promote allergic inflammation [22,37], 6 sub-clusters were identified (Fig. S6C and D). Consistent with the reports of previous studies, a group of CD8^+^ Tem cells was identified that expressed uniquely elevated levels of S100A4 and granzyme K (GZMK) transcripts (Fig. 8N and Fig. S6D). After exposure to the allergen, the accumulation of S100A4^+^GZMK^+^CD8^+^ Tem cells increased compared to that in the control group (Fig. 8O). Interestingly, the anti-TL1A treatment significantly decreased the abundance of S100A4^+^GZMK^+^CD8^+^ Tem cells (Fig. 8O). The flow cytometry results validated the decrease in the number of S100A4^+^CD62L^low^CD44^hi^ Tem cells (Fig. 8P), confirming the scRNA-seq findings.

## Discussion

Epithelial alarmins, including IL-25, IL-33, and TSLP, have garnered increasing attention owing to the successful outcomes of related clinical studies and drug trials [5,38]. As primary initiators of allergic immune responses, these alarmins exert effects upstream of asthma inflammatory cascades, demonstrating promising therapeutic potential for the treatment of broader asthma phenotypes [39]. TL1A, a newly recognized epithelial alarmin, exerts pleiotropic effects that are mediated via DR3 activation [11]. The present study extends the therapeutic potential of this paradigm by demonstrating elevated expression levels of TL1A in asthmatic serum and sputum samples that correlated with poor disease control, blood eosinophilia, and declining lung function. The murine models further revealed early TL1A elevation in serum and BAL samples post-allergen challenge, with progressive increases coinciding with repeated allergen exposure, airway inflammation, and remodeling. We posit that TL1A may play a dual role in the asthmatic inflammatory network: it may function as an early alarm signal under specific conditions, while in sustained inflammation, it acts more as a secondary mediator that amplifies and maintains the inflammatory cascade. This understanding may help guide more precise timing and patient selection for TL1A-targeted therapies.

Despite the restricted cellular distribution of DR3 and the many studies traditionally implicating T cells and innate lymphoid cells (ILCs) as the primary TL1A targets in allergic asthma [12,13,18], macrophages were identified as both the main immune cellular source and effector target of TL1A within allergic pulmonary microenvironments. Consistent with previous reports [10,18], DR3 expression persisted on macrophages at baseline and poststimulation. Crucially, myeloid-cell-specific genetic deletion of *Tnfsf15* attenuated airway inflammation, eosinophil infiltration, and type 2 cytokine production, establishing macrophage-derived TL1A as an independent contributor to asthma pathogenesis. This coincidence of source and target suggests that TL1A likely operates through 2 concurrent modes. TL1A from multiple cellular origins (including epithelial cells and macrophages) can act directly on macrophage DR3 receptors, activating downstream signaling pathways, thereby inducing the expression of inflammatory factors. Simultaneously, secreted TL1A can diffuse within the microenvironment and act on neighboring DR3-expressing immune cells, such as CD4^+^ T cells, CD8^+^ T cells, and type 2 ILCs, promoting their differentiation into pathogenic subsets like Th2 cells and cytotoxic effectors, thereby perpetuating and amplifying the broader inflammatory cascade. In this study, myeloid-specific KO of *Tnfsf15* eliminated both the source of TL1A and its secondary amplifying effect, leading to significant attenuation of airway inflammation. This reveals the dual advantage of anti-TL1A therapy in simultaneously intervening in both innate and adaptive immunity.

TL1A has been reported to modulate macrophage function through reactive oxygen species promotion and pro-inflammatory mediator production [7,33]. This function is underpinned by its canonical signaling through DR3, which activates both NF-κB and MAPK pathways, a foundational mechanism established in T cells and ILCs [31]. This DR3-dependent signaling axis is a potent driver of inflammatory gene expression, thereby orchestrating leukocyte recruitment and directly inducing the production of chemokines [40]. Building upon this established paradigm, our transcriptomic analysis of lung tissue from myeloid-cell-specific *Tnfsf15* KO mice revealed a profound suppression of CCL8, a chemokine mainly produced by macrophages that plays a pivotal role in eosinophilic inflammation via CCR8 binding [26,28]. Subsequent CCR8 blockade in the animal models markedly attenuated TL1A-mediated airway inflammation following exogenous allergen challenge, confirming the CCL8–CCR8 axis as a critical and specific downstream effector of TL1A in asthma. Furthermore, our pharmacological inhibition experiments demonstrated that soluble TL1A activates CCL8 through MAPK (p38), PI3K, and NF-κB signaling cascades, revealing a multipathway regulatory mechanism underlying allergic inflammation [11,30,34,35]. It should be noted that the *Tnfsf15* KO in this study, mediated by the *Lyz2*-iCre driver system, occurs in all LysM-expressing myeloid cells, including macrophages and neutrophils, among others. While we confirmed effective TL1A protein knockdown in sorted lung F4/80^+^ macrophages (Fig. 5C), and neutrophils themselves are not major producers of TL1A, this study could not fully rule out contributions from neutrophils or other LysM^+^ myeloid cell types to the observed phenotype. Future studies employing more specific macrophage-subset Cre driver lines will help further dissect these contributions.

The upstream inducers of TL1A in the airway epithelium and macrophages in allergic inflammation remain poorly characterized. TNF-α and TGF-β are known to induce TL1A in airway epithelial cells [30,31], and this study confirms these findings and further demonstrates that poly(I:C) transfection (mimicking viral RNA) activates RIG-I/MDA5 to up-regulate TL1A, reinforcing its epithelial alarmin function. In macrophages, which exhibit phenotypic plasticity beyond the traditional M1/M2 paradigms [41], TL1A expression is inducible by TSLP or IL-33. Notably, IL-33/IL-2 costimulation, which has been shown to generate pathogenic macrophages M(IL-33+IL-2) in asthma [32], synergistically enhanced TL1A production in this study, suggesting that macrophage TL1A induction is dependent on combinatorial inflammatory signals rather than a single stimulus.

Targeted therapy has the potential to revolutionize asthma management, and several TL1A monoclonal antibodies are currently undergoing preclinical evaluations to verify their efficacy and safety [14–16]. To elucidate the immunological impact of TL1A-neutralizing antibody therapy on pulmonary immunity, classical murine asthma models were established, and varying doses of an anti-TL1A antibody were administered. Beyond modulating the Th1/Th2/Th17 cell balance, the anti-TL1A intervention appeared to alter the distribution of CD8^+^ T-cell subsets, thereby reducing pathogenic CD8^+^ T-cell populations. As reported in this study and corroborated by the existing literature, the abundances of specific Tem cell subsets are elevated in asthmatic microenvironments and have been shown to contribute to disease progression [34,37]. Sustained allergen exposure diminishes interferon gamma (IFN-γ) production in CD8^+^ Tem cells, thereby disrupting the Th1/Th2 equilibrium [22]. Under allergen exposure conditions, GZMK^+^CD8^+^ Tem cells facilitate eosinophil recruitment and type 2 effector activation via the complement pathway, independently exacerbating airway inflammation [37]. In the present study, the proportion of S100A4^+^GZMK^+^CD8^+^ Tem cells was significantly diminished after blocking TL1A, suggesting that TL1A may affect the proliferation and differentiation of CD8^+^ T cells to amplify allergic inflammation. These findings expand the mechanistic understanding of the means by which anti-TL1A therapeutic agents attenuate allergic airway pathology.

This study had some limitations. First, it was not possible to determine the correlation between sputum TL1A levels and clinical parameters in patients with asthma, and a substantial proportion of the induced sputum samples had expression levels below the detection threshold of the assay. This technical limitation likely stems from suboptimal sputum processing protocols and quality control measures. However, it is noteworthy that the majority of these undetectable samples were from individuals with mild disease or healthy subjects, which may to some extent reflect the expression trend of TL1A. Therefore, while our data indicate elevated TL1A in the sputum of a subset of patients with asthma (particularly those with more severe disease), the conclusions regarding its utility as a sputum biomarker must be tempered by these analytical constraints. Future studies employing ultrasensitive immunoassays, optimized sputum processing, or analysis of BAL are warranted to conclusively define the role of sputum TL1A. Second, technical limitations of scRNA-seq, combined with the inherently low transcriptional abundance of TL1A, limited the resolution of epithelial cells and ILCs, as well as the identification of distinct macrophage subsets with elevated TL1A expression. Consequently, the role of TL1A in these populations could not be definitively assessed. Future high-resolution single-cell atlases with enhanced sensitivity will better characterize TL1A-high macrophage subsets and elucidate the functional impact of TL1A blockade on adaptive immune responses, including T-cell responses.

**Fig. 9.**
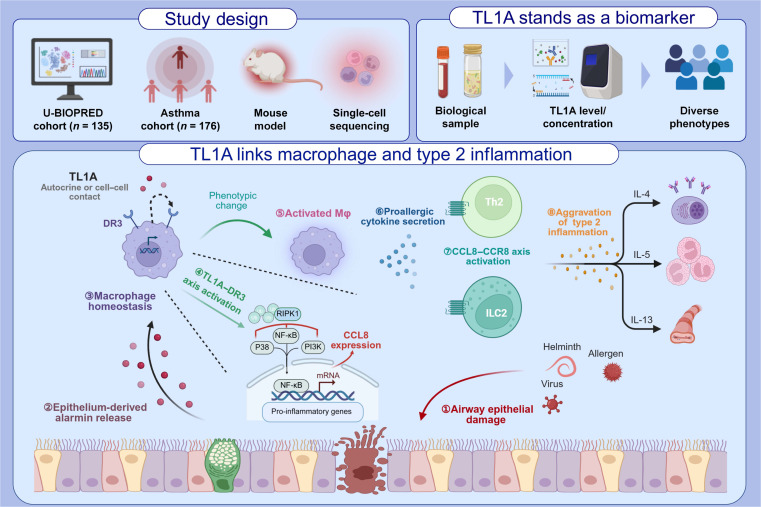
Main findings of the study. The TL1A level is elevated in the serum and sputum of asthmatic patients and is correlated with asthma progression. TL1A affected macrophage homeostasis and induced type 2 inflammation via the CCL8/CCR8 pathway. IL, interleukin; Th2, T helper 2 cell; ILC2, type 2 innate lymphoid cell; TL1A, TNF-like ligand 1A; DR3, death receptor 3; RIPK1, receptor-interacting protein kinase 1; NF-κB, nuclear factor kappa B; PI3K, phosphatidylinositol 3-kinase; CCL, chemokine C-C motif ligand; CCR, chemokine C-C motif receptor.

Collectively, this study establishes TL1A as a biomarker and therapeutic target in asthma, revealing its role in driving macrophage-dependent inflammation via the CCL8/CCR8 axis (Fig. 9). Our findings further highlight the TL1A-mediated immunoregulation of CD4^+^ and CD8^+^ T-cell populations, providing a mechanistic basis for TL1A-targeted therapies and advancing prospects for precision asthma management. Looking forward, given the potential implication of TL1A in severe asthma and irreversible lung function decline, investigating its source-specific impact on airway remodeling in chronic asthma models represents a critical next step. We propose that anti-TL1A treatment may promote a coordinated recalibration of both innate and adaptive immunity—a compelling hypothesis warranting further validation in well-powered studies designed to delineate the underlying T-cell dynamics.

## Materials and Methods

### Cell culture and treatment

The human airway epithelial cell line HBE was obtained from the Shanghai Fuheng Cell Center. Cells were cultured in Keratinocyte Medium (ScienCell) supplemented with 1% Keratinocyte Growth Supplement (ScienCell) and then incubated at 37 °C in a humidified incubator with a 5% CO_2_ environment. The transfection was performed with plasmid/small interfering RNA (GenePharm, Shanghai, China) using EndoFectin-Max (GeneCopoeia) according to the manufacturer’s instructions. The recombinant proteins, small-molecular compounds, or inhibitors were purchased as indicated: recombinant mouse IL-13, IL-33, and TSLP protein (MCE); recombinant human IL-2, IL-4, TNF-α, and IFN-γ protein (Abbkine); poly(I:C) (Selleck); lipopolysaccharide (Sigma-Aldrich); IL-1β (Thermo Fisher); and small-molecule inhibitors BAY11-7082, LY294002, PD98059, SB203580, and SP600125 (Selleck).

BMDMs were obtained by flushing the bone marrow from mouse leg bones using a syringe. After removing the red blood cells, it was centrifuged, the supernatant was discarded, and resuspended with Dulbecco’s modified Eagle medium complete culture medium (penicillin 100 U/ml, streptomycin 100 U/ml, and macrophage colony-stimulating factor [M-CSF] 20 ng/ml). The solution was changed on the third and fifth days, and BMDMs were obtained after 7 d of induction. Human THP-1 cells were obtained from the American Type Culture Collection. Phorbol myristate acetate-activated THP-1 cells were used as human macrophages. The cells were cultured at 37 °C under 5% CO_2_ in RPMI 1640 medium supplemented with 10% fetal bovine serum (FBS; Invitrogen-Gibco). Primary human monocyte-derived macrophages were isolated by centrifugation of discarded normal blood from healthy subjects. Monocytes were enriched from freshly isolated peripheral blood-derived mononuclear cells by positive selection on CD14 microbeads followed by separation on MACS columns. Macrophages were obtained from human monocytes after 10 d of culture in RPMI 1640 medium supplemented with 10% FBS, 1% sodium pyruvate, 0.1% β-mercaptoethanol, and human M-CSF (20 ng/ml).

### Animals, model establishment, and antibody interventions

C57BL/6 background *Tnfsf15*-KO mice (strain no. T011739), *Tnfsf15*-flox mice (strain no. T010024), and *Lyz2*-icre mice (strain no. T003822) were purchased from GemPharmatech. The mice were maintained in a standard laboratory animal facility for 1 week prior to the initiation of the experiments. In the OVA-induced asthma models, the mice were injected intraperitoneally with 20 μg of OVA (Sigma-Aldrich) and 2 mg of aluminum hydroxide to promote sensitization. During the challenge stage, the mice were placed in a stimulation chamber and administered a 1.5% OVA saline solution for aerosol inhalation over a period of 30 min daily. The control group received only injections and atomization with phosphate-buffered saline (PBS). For the HDM group, mice were exposed to HDM extracts (Greer Laboratories). While the experimental group was sensitized with intranasal instillation of 50 μl of the HDM suspension for 28 d over 4 consecutive weeks, the control group was sham sensitized with PBS. To deplete the population of pulmonary macrophages, the mice underwent allergen sensitization as described above before being subsequently treated intratracheally with 30 μl of either clodronate-encapsulated liposomes (Yeasen) or empty liposomes encapsulating as a control prior to the OVA challenge. BAL, serum samples, and the lungs of the mice were collected 24 h (unless specified otherwise) after the final nebulization. BAL was performed as previously described [42]. The rTL1A (BioLegend), anti-immunoglobulin G, anti-CCR8 (BioLegend), and anti-TL1A (Bio X Cell) groups were treated with neutralizing antibodies before each challenge.

### Immunoblotting

Briefly, cells were homogenized in radioimmunoprecipitation assay lysis buffer with phenylmethanesulfonyl fluoride. Total protein was separated by 10% sodium dodecyl sulfate–polyacrylamide gel electrophoresis and transferred to polyvinylidene fluoride membranes. Then, 3% bovine serum albumin was used to block nonspecific sites for 1 h. After that, the membranes were incubated with primary antibodies overnight at 4 °C and incubated with secondary antibody for 1 h the next day. The primary antibodies are listed as follows: anti-TL1A (1:1,000, Affinity), anti-F4/80 (1:1,000, Huabio), anti-β-actin (1:10,000, Huabio), and anti-glyceraldehyde-3-phosphate dehydrogenase (1:10,000, Huabio). After washing with Tris-buffered saline plus 0.1% Tween-20 again, the membranes were subjected to chemiluminescence by using an enhanced chemiluminescence substrate.

### Histology and immunofluorescence

The lung of each mouse was embedded in paraffin according to standard procedures. Sections (5 μm) were mounted on slides for histological or immunofluorescence analysis. Hematoxylin and eosin staining was used to evaluate changes in lung morphology. Mucus secretion was assessed by periodic acid–Schiff staining. Masson staining was used for staining collagen deposition. Immunofluorescence staining was performed on paraffin sections started by deparaffinization through ethanol series.

### Micro-CT

The mice were anesthetized and subsequently placed in a micro-CT system (Quantum GX2) for in vivo data acquisition.

### Clinical sample collection

Human sputum and serum samples were collected from outpatients with asthma (*n* = 128) and healthy controls (*n* = 48) at the First Affiliated Hospital of Shandong First Medical University and from community-based healthy volunteers. Spontaneously expectorated morning sputum samples were weighed and diluted with 4 volumes of pre-cooled PBS containing 0.1% dithiothreitol. The mixture was rotated until liquefied and passed through nylon mesh to remove large debris. The filtrate was centrifuged at 400 × g for 10 min at 4 °C, and the supernatant was stored at −80 °C. The cell pellet after wash was lysed in 1 ml of TRIzol reagent and stored at −80 °C. Asthma was diagnosed according to the Global Initiative for Asthma (GINA) guidelines (updated in 2024), which are based on the presence of symptoms such as cough, shortness of breath, wheezing, or chest tightness and variable airflow limitation. The healthy participants had no history or current symptoms of chronic respiratory, metabolic, and allergic diseases or other conditions that could have affected the outcomes. Severe asthma, defined as treatment per GINA step 5 or having uncontrolled disease despite treatment in GINA step 4, was included. The experiments involving human subjects were approved by the local ethics committee (ethics review numbers: 2021-S923 and 2022-S011), and the study was conducted in accordance with the principles of the Declaration of Helsinki.

### Screening and analysis of public RNA profiles

Gene set enrichment (GSE) datasets pertaining to transcriptional profiles in patients with asthma (GSE69653, GSE69683, GSE76262, GSE74075, GSE184433, and GSE27011) and murine models (GSE184433) were screened and selected from the Gene Expression Omnibus database. The profile graph function from the GEO2R program was used to obtain expression values and generate the expression profile graphs for the different groups. CIBERSORT was used to determine the relative frequencies of immune cells in each sample. The normalized gene expression data were transformed into immune cell information by the CIBERSORT deconvolution algorithm. Linear regression analysis was used to analyze the correlation between TL1A expression and immune cells.

### Flow cytometry

Single-cell suspensions prepared from mouse lung tissue were blocked with 5% FBS in PBS for 30 min. To label cell surface proteins, the cells were incubated with the following corresponding flow cytometric antibodies: mouse cluster of differentiation (CD)3e, CD4, CD8a, CD11b, CD11c, CD25 (all from MultiSciences), CD44, CD45, CD62L, CD170 (all from BioLegend), and TL1A (Tandys1a, Invitrogen). The collected cells were incubated in antibody solution for 30 min at 4 °C, and isotype IgG was used as a control.

To label intercellular proteins, including S100A4 (Abcam), forkhead box protein P3 (Foxp3; MultiSciences), IL-4 (MultiSciences), and interleukin 17A (IL-17A, MultiSciences), cells were first fixed with intracellular fixation and permeabilization buffer A (MultiSciences) for 15 min. After washing, the cells were resuspended and incubated on ice with the corresponding flow cytometric antibodies (anti-S100A4, anti-Foxp3, anti-IL-4, and anti-IL-17A) and permeabilization buffer B (MultiSciences) for 30 min at 4 °C. The cells were subsequently washed with Flow Cytometry Staining Buffer (MultiSciences) and collected after centrifugation at 3,000 rpm for 5 min at 4 °C. Finally, the cells were analyzed using a FACSCalibur or FACSAria III (BD Biosciences) flow cytometer and the FlowJo 10.8.1 software. After gating live, single CD45^+^ cells from total lung cells were separated into CD4^+^ T cells (CD3^+^CD4^+^), CD8^+^ T cells (CD3^+^CD8^+^), B cells (CD3^−^CD19^+^), alveolar macrophages (CD11c^+^CD170^+^), eosinophils (CD170^+^CD11c^−^), and pulmonary macrophages (CD11c^+^F4/80^+^). For lymphoid cell analysis, we used CD44^high^CD62L^low^, CD44^high^CD62L^high^, CD4^+^IFN-γ^+^, CD4^+^IL-4^+^, CD4^+^IL-17A^+^, and CD4^+^CD25^+^Foxp3^+^ to identify Tem, central memory T, Th1, Th2, Th17, and Treg cells, respectively.

### Cell sorting

F4/80^+^ cells in lung tissue were sorted using a mouse F4/80 positive selection kit (StemCell). The following steps were performed according to the reagent instructions with appropriate adjustment: The lung tissue was harvested and minced in digestion medium (RPMI 1640 containing collagenase/hyaluronidase and DNase I), followed by incubation at 37 °C for 20 min with shaking. The digested tissue was then mechanically dissociated and sequentially filtered through nylon mesh to obtain a single-cell suspension. After centrifugation, red blood cells were lysed and the remaining cells were washed, centrifuged again, and finally resuspended in 1 ml of PBS containing 2% FBS. Unspecific binding was prevented using 50 μl of FcR-PolyBlock, followed by 60 μl of selection cocktail. Magnetic RapidSpheres were vortexed for 1 min before 80 μl was added to the isolation mix for 5 min. The reaction was resuspended and inserted into a magnet for 5 min before the purified macrophage suspension was carefully decanted into a new tube.

### Single-cell RNA sequencing

scRNA-seq was performed to characterize the single-cell transcriptomic landscape of the lung tissue. Single-cell libraries were prepared using the BD Rhapsody Single-Cell Analysis System (BD Biosciences) according to the manufacturer’s protocol. Libraries were sequenced using multiple runs on an Illumina NextSeq platform. Cell types were annotated using a combination of scRNA-seq cell annotation software, SingleR, and gene expression markers.

Following sequencing, the raw FASTQ files were processed through an analysis pipeline for sequence alignment using a reference genome and transcriptome annotation. This pipeline produced a unique molecular identifier (UMI) count matrix, which was subsequently analyzed with Seurat. Quality control was performed by filtering out low-quality cells and potential multiplets: cells with UMI or gene counts deviating beyond mean ± 2 standard deviations (SDs) were excluded, as were cells where mitochondrial genes accounted for >50% of total counts. After this filtering, high-quality single cells were retained for downstream analysis. Library size normalization was carried out using the NormalizeData function in Seurat, applying a log-normalization procedure where counts per cell were scaled by a factor of 10,000 and log-transformed. Highly variable genes were identified using the FindVariableGenes function. To correct for batch effects, the mutual-nearest-neighbor method was applied. Cell clustering was performed using the shared nearest neighbor algorithm, implemented through the FindNeighbors and FindClusters functions in Seurat with default parameters. Each major cell type was also reclustered independently. Dimensionality reduction and visualization were achieved using t-distributed stochastic neighbor embedding via the RunTSNE function.

Cluster-specific marker genes were identified with the FindMarkers function, which detects genes positively enriched in each cluster compared to all others. Differential expression analysis between conditions was conducted using FindMarkers. Gene Ontology and Kyoto Encyclopedia of Genes and Genomes pathway enrichment analyses of differentially expressed genes were performed in R based on the hypergeometric distribution.

To analyze ligand–receptor interactions from the scRNA-seq data, we applied CellPhoneDB. A gene was considered expressed if nonzero read counts were detected in at least 10% of the cells within a given cluster. Cell–cell communication networks were then constructed by connecting pairs of cell types where one expressed a specific ligand and the other expressed its cognate receptor. The resulting interaction networks were visualized using the Circlize packages.

### RNA isolation and RT-PCR analysis

Total RNA was extracted using the RNA Fast 200 RNA Extraction kit (Fastagen) or a TRIzol RNA extraction kit (Invitrogen). After quantification using spectrophotometry, 1 μg of RNA was used to synthesize complementary DNA by using an Evo M-MLV Mix Kit with gDNA Clean for qPCR (Accurate Biology). Reverse transcription quantitative PCR was performed using the SYBR Green PCR Master Mix (Accurate Biotechnology). All samples were run in triplicate, and the mean values were used for quantification.

### Enzyme-linked immunosorbent assays

All cytokines were measured using commercial ELISA kits according to manufacturers’ instructions. The ELISA kits used were as follows: mouse CCL8 (ZCI BIO), mouse CXCL-2 (MultiSciences), mouse CXCL-10 (MultiSciences), mouse IFN-γ (MultiSciences), mouse IL-4 (MultiSciences), mouse IL-6 (MultiSciences), mouse IL-13 (MultiSciences), IFN-γ (MultiSciences), mouse total IgE (Jianglaibio), mouse TL1A (ZCI BIO), and human TL1A (ZCI BIO, Catalog No. ZC-35762, had a stated lower limit of detection of 5 pg/ml, as per the manufacturer’s specifications).

### RNA-seq analysis

After modeling, the lung tissues of each KO and control mouse were separated and stored at −80 °C for RNA-seq. RNA-seq analysis was carried out by Sinotech Genomics Co., Ltd. (Shanghai, China).

### Statistical analysis

All experiments were repeated at least 3 times to provide robust data for analysis. Intergroup comparisons were conducted using an unpaired *t* test for parametric parameters and the Mann–Whitney *U* test for nonparametric parameters and one-way analysis of variance with Tukey’s post hoc testing to correct for multiple comparisons. Baseline laboratory parameters and clinical variables were analyzed separately using parametric or nonparametric tests according to their distribution. For sequencing data comparisons, the nonparametric Mann–Whitney *U* test or Kruskal–Wallis test was employed. All *P* values <0.05 were considered statistically significant. Statistical analyses were conducted using the GraphPad Prism software (version 8.0; GraphPad Software). Unless otherwise stated, data are presented as mean ± SD from at least 3 independent experiments. The sample size (*n*) for each experiment, denoting the number of biological replicates, is provided in the figure legends. Data collection and analysis for all experimental studies, including histology scoring, flow cytometry, and image quantification, were performed by investigators blinded to group allocation. For public dataset analyses, original sample sizes are cited.

## Ethical Approval

Written informed consent was obtained from all subjects, and this research was conducted in accordance with the Declaration of Helsinki. The study was approved by the ethical committee of the First Affiliated Hospital of Shandong First Medical University (ethics review numbers: 2021-S923 and 2022-S011).

## Data Availability

The data that support the findings of this study are available from the corresponding author upon reasonable request.
