# Supplementary material for: Integrated Single-Cell Profiling Reveals TL1A as a Biomarker and Driver of Type 2 Inflammation via Macrophage-Dependent Immunoregulation in Asthma
Source: Research (Wash D C). 2026 Apr 9;9:1190. doi: 10.34133/research.1190 (PMC13062487; doi:10.34133/research.1190)
Supplement: Supplementary 1 — Table S1 Figs. S1 to S6 [file research.1190.f1.zip › SM.docx]

**Figure S1. Immune infiltration analysis of TL1A. (A)** CIBERSORT algorithm analysis of immune cell infiltration panorama. For each cluster, we calculated the median of the absolute score of the 22 cell types given by the CIBERSORT in each cohort. Immune cluster-specific cell-type median scores were used in an unsupervised clustering using maximum linkage and the ward.D2 method. The heatmap obtained allows to visualize which cell types are enriched across the immune clusters. Immune clusters are annotated on the top and bottom of the heatmap. **(B)** Boxplot shows the immune cell infiltration difference between the control and Asthma groups. Blue represents the control group; red represents the asthma group. **(C)** Boxplot shows the immune cell infiltration difference between the mild and severe asthma groups. Blue represents the mild group; red represents the severe group. **(D-G)** Correlation between TL1A gene expression level and immune cell (eosinophils, activated mast cells, plasma cell, and dendritic cells) infiltration. Data are presented as the mean ± SD. CIBERSORT analysis was performed on transcriptomic data from public cohorts (sample sizes as per original datasets). Correlation analyses included all available samples. Analyses were not blinded due to their computational nature.

**Figure S2. TL1A expression and secretion in HDM-induced asthma model.** **(A)** Western blot analysis of TL1A protein expression in the lung tissues in the wild type and HDM-induced model groups. **(B-C)** Comparison of TL1A concentrations in serum/BAL samples in each group. Data are presented as the mean ± SD from n = 6 mice per group, representative of two independent experiments. All measurements (ELISA, cell counts, scoring) were performed blinded to group identity.

**Figure S3. Myeloid cell-specific *Tnfsf15*-nockout resulted in the attenuation of allergic airway inflammation in HDM-induced asthma model. (A)** Representative HE staining among the different groups, and quantification of the airway inflammation score. **(B)** Representative PAS staining among the different groups, and quantification of the airway mucus score. **(C-D)** Lung eosinophil or neutrophil counts, as determined by flow cytometry. **(E)** Total cell counts in BAL. **(F-J)** Concentrations of IL-4, IL-13, IFN-γ, CXCL2 or CXCL10 in BAL. Data are presented as the mean ± SD from n = 6 mice per group, representative of two independent experiments. All measurements (ELISA, cell counts, scoring) were performed blinded to group identity.

**Figure S4. The TL1A-treated model mice induced by HDM exhibited increased CCL8 expression and aggravated lung inflammation. (A)** HE staining of lung tissue after allergen challenge and rTL1A or anti-CCR8 interventions. **(B)** Total cell numbers in BAL samples. **(C-D)** Concentrations of CCL8, IL-4, and IL-13 in BAL samples from the different groups. Data are presented as the mean ± SD from n = 6 mice per group, representative of two independent experiments. All measurements (ELISA, cell counts, scoring) were performed blinded to group identity.

**Figure S5. Analysis of CCR8 expression cell subpopulations.** **(A)** Characteristics of single-cell CCR8 expression. **(B)** CCR8 expression characteristics of T cell subsets. Data are derived from scRNA-seq analysis pooled from n = 4 mice (as in Figure 8). Bioinformatics analysis was not blinded.

**Figure S6. Analysis of CD8^+^ T cell subpopulations in the different treatment groups.** **(A)** Characteristics of single-cell TNFRSF25 expression. **(B-C)** The distribution of CD8^+^ T cell subsets in each group. **(D)** Gene expression characteristics of seven CD8^+^ T cell subsets. Data are derived from scRNA-seq analysis of CD8^+^ T cells pooled from n = 4 mice (as in Figure 8). Bioinformatics analysis was not blinded, but downstream validation by flow cytometry was performed blinded.
