# Supplementary material for: Integrated Single-Cell Profiling Reveals TL1A as a Biomarker and Driver of Type 2 Inflammation via Macrophage-Dependent Immunoregulation in Asthma
Source: Research (Wash D C). 2026 Apr 9;9:1190. doi: 10.34133/research.1190 (PMC13062487; doi:10.34133/research.1190)
Supplement: Supplementary 1 — Table S1 Figs. S1 to S6 [file research.1190.f1.zip › Table S1.docx]

| **Table S1. Clinical characteristics of the asthma and healthy control groups** | | |
| --- | --- | --- |
|  | Asthma (n = 128) | Control (n = 48) |
| Sex, n (male/female) | 60/68 | 22/26 |
| Age (years) | 50.5±14.13 | 48.31±12.88 |
| Height (cm) | 164.66±9.32 | 166.35±9.14 |
| BMI (kg/m^2^) | 24.63±4.70 | 23.74±4.12 |
| WBC count (*10^9^) | 7.72±1.91 | 6.01±1.17 |
| NEU count (*10^9^) | 4.48±1.65 | 3.51±1.06 |
| EOS count (*10^9^) | 0.51±0.28 | 0.09±0.05 |
| IgE (IU/mL) | 267.59±290.60 | 20.16±18.24 |
| FVC | 100.33±19.41 | 107.61±12.36 |
| FEV1/FVC | 67.49±12.85 | 83.12±5.57 |
| FEV1% | 83.52±25.06 | 105.24±8.69 |

Values in the table are reported as frequencies or as means ± standard deviations.

Abbreviations: BMI, body mass index; WBC, white blood cell; NEU, neutrophil; EOS, eosinophil; IgE, immunoglobulin E; FVC, forced vital capacity; FEV1, forced expiratory volume in one second
