# Supplementary figures and images for: Integrated Single-Cell Profiling Reveals TL1A as a Biomarker and Driver of Type 2 Inflammation via Macrophage-Dependent Immunoregulation in Asthma
Source: Research (Wash D C). 2026 Apr 9;9:1190. doi: 10.34133/research.1190 (PMC13062487; doi:10.34133/research.1190)

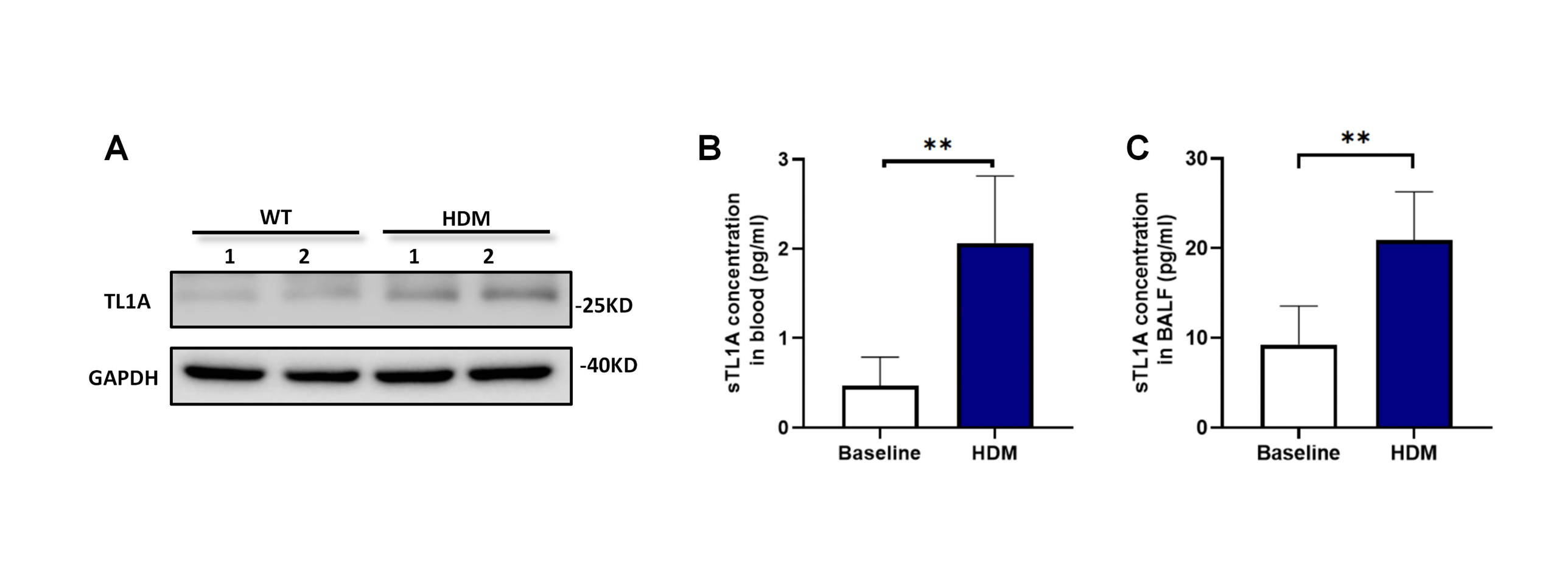

Supplement: Supplementary 1 — Table S1 Figs. S1 to S6 [file research.1190.f1.zip › Figure S2.jpg]
